# Development and validation of a pen side test for Rift Valley fever

**DOI:** 10.1371/journal.pntd.0007700

**Published:** 2019-09-11

**Authors:** Catherine Cêtre-Sossah, Aurélie Pédarrieu, Mikael Juremalm, Petrus Jansen Van Vuren, Alejandro Brun, Ahmed Bezeid Ould EL Mamy, Jean-Michel Héraud, Claudia Filippone, Jean-Pierre Ravalohery, Hassan Chaabihi, Emmanuel Albina, Laure Dommergues, Janusz Paweska, Eric Cardinale

**Affiliations:** 1 ASTRE, Univ Montpellier, CIRAD, INRA, Montpellier, France; 2 CIRAD, UMR ASTRE, Sainte‐Clotilde, La Réunion, France; 3 Boehringer Ingelheim Svanova, Virdingsallé, Uppsala, Sweden; 4 Centre for Emerging Zoonotic and Parasitic Diseases, National Institute for Communicable Diseases, Sandringham-Johannesburg, South Africa; 5 INIA-CISA, Valdeolmos, Madrid, Spain; 6 ONARDEL, service de pathologies infectieuses, Nouakchott, Mauritania; 7 Virology Unit, Institut Pasteur de Madagascar, Antananarivo, Madagascar; 8 Agate bioservices, Bagard, France; 9 CIRAD, UMR ASTRE, Petit Bourg, Guadeloupe, France; 10 CoopADEM - GDS Mayotte, Coconi, Mayotte, France; Saudi Ministry of Health, SAUDI ARABIA

## Abstract

**Background:**

Rift Valley fever (RVF) is one of the main vector borne zoonotic diseases that affects a wide range of ruminants and human beings in Africa and the Arabian Peninsula. A rapid and specific test for RVF diagnosis at the site of a suspected outbreak is crucial for the implementation of control measures.

**Methodology/Principal findings:**

A first-line lateral flow immunochromatographic strip test (LFT) was developed for the detection of the nucleoprotein (N) of the RVF virus (RVFV). Its diagnostic performance characteristics were evaluated using reference stocks isolates recovered from different hosts and in geographic regions mimicking clinical specimens and from known RVF negative serum samples. A high level of diagnostic accuracy (DSe (35/35), DSp (167/169)) was observed, including the absence of cross-reactivity with viruses belonging to different genera.

**Conclusion/Significance:**

The fact no specialized reagents and laboratory equipment are needed, make this assay a valuable, first-line diagnostic tool in resource-poor diagnostic territories for on-site RVFV detection, however the staff require training.

## Author summary

Rift Valley fever (RVF) is a viral disease that affects a wide range of animals and human beings in Africa and the Arabian Peninsula involving low case fatality rates. A rapid and specific test for RVF diagnosis at the site of a suspected outbreak is crucial for the implementation of control measures. Here, we report the development and the evaluation of the diagnostic performance characteristics of a pen-side test found to be a highly accurate and valuable first-line diagnostic tool for on-site RVF detection.

## Introduction

Rift Valley fever (RVF) is an emerging mosquito-borne disease that affects a wide range of animals and human beings in Africa and the Arabian Peninsula. It is caused by the mosquito-borne Rift Valley fever phlebovirus (RVFV) that belongs to the genus *Phlebovirus* in the family *Phenuiviridae* of the order *Bunyavirales* [1]. First identified in the Great Rift Valley in Kenya in 1930 and initially confined to the African continent, it subsequently spreads to Madagascar, the archipelago of Comoros, and the Arabian Peninsula [2]. There is a growing concern that RVFV will extend its current range due to the wide variety of mosquito species able to transmit to several mammal hosts. This includes species distributed in countries outside Africa and Arabian Peninsula where RVFV is not yet known to circulate despite the environmental factors driving and favoring its circulation [3–6].

Recent outbreaks of RVF in Mayotte, Niger, Uganda and Sudan involving human deaths and characterized by mass abortion and high mortality rates of neonates in the ruminant population raised international interests in improving diagnostic and vaccine control strategies [7–10].

When investigating disease outbreaks in animals, the earlier the clinical signs of disease are recognized by the farmer and the earlier the clinical diagnosis is confirmed by laboratory tests, along with rapid reporting to the relevant veterinary authorities, the better the disease will be controlled. Success partly relies on sending samples to a reference laboratory to test for the presence of RVFV, with high levels of viral particles in the serum during the acute phase of the disease.

The availability of a ‘pen-side’ diagnostic test would have the advantage of providing additional support for the medical clinical judgment in the first instance and could reduce the time needed to confirm the test results in secondary cases of the disease. The use of rapid diagnostic tests that can be conducted in the field, at the site of the outbreaks, where the infected human and animal populations are, will therefore facilitate earlier and more effective disease control. Conventional techniques for the diagnosis of RVF include virus isolation, detection of specific IgM or IgG antibodies, and detection of RVFV specific nucleic acids. Enzyme-linked immunosorbent assays (ELISA), based on whole virus antigens or the recombinant nucleocapsid protein N have been extensively validated for the serodiagnosis of RVF [11–12]. Conventional and real-time reverse transcriptase polymerase chain reaction (RT-PCR) assays are currently the most rapid and sensitive tests for the detection and quantification of RVFV during outbreaks [13–17]. Methods based on next generation sequencing (NGS) approaches [18] or colorimetry [19], TaqMan array cards [20] have been recently developed but most of these techniques are expensive and require dedicated trained personnel and equipped biosafety level 3 laboratories that are often not available in the areas where the disease occurs. There is thus a need for a simpler, inexpensive and reliable pen-side test to facilitate prompt and accurate field diagnosis. Lateral flow tests (LFT) also known as immuno-chromatographic strips are rapid, single-use, one-step test devices able to detect at point of care the presence of an analyte in a liquid sample, flowing along a membrane strip encased in a protective plastic frame. The result can be easily seen with the naked-eye (test line and control line). Good examples have already been published for the detection of Ebola, rabies viruses and visceral leishmaniasis [21–23]. In this study, we evaluated the performances of a robust and rapid test for the detection of Rift Valley fever virus that should be a useful diagnostic tool for RVF control as it will rapidly detect the first outbreak thereby limiting disease spread through appropriate surveillance in the framework of a disease management program in developing countries.

## Materials and methods

### Ethics statement

No endangered or protected species were involved in the surveys. Farmers in each zone gave their verbal consent to be included in the study. Consent for blood sampling on a herd was obtained from its owner verbally after information was provided in French (official language) or Shimaore, Malagasy (local languages). Animals sampled by qualified veterinarians were bled without suffering. The animal serum samples that originated from mainland France, Reunion Island, Tunisia and the Union of Comoros were collected during either a cross-sectional or a longitudinal survey as described previously [24–25]. Animal serum samples from Mayotte were collected under a national disease surveillance system SESAM with the approval of the Direction of Agriculture, Food and Forestry (DAAF) of Mayotte [26]. Animal serum samples from Madagascar were collected in collaboration with the Malagasy veterinary services [27].

### RVFV N specific monoclonal antibodies used in the LFT

#### Plasmid, cloning and generation of recombinant baculoviruses

The N nucleoprotein and the G_N_/Gc glycoprotein genes of the RVF ZH-548 strain isolated from a human infection during the 1977 outbreak in Egypt [28] were used in this study. The coding sequence of the N recombinant nucleoprotein amplified using the primer N Rift-5’KpnI (5’- nnnnGGTACCATGGACAACTATCAAGAGC-3’) and the primer N Rift-3’BglII (5’-nnnnAGATCTTTAGGCTGCTGTCTTGTAAGC-3’) was inserted in the KpnI-BglII restriction sites of the plasmid pΔPhC3T (Agate bioservices, Bagard, France) to obtain the transfer vector named pΔPhC3T-N. The vector contains the N coding sequence downstream of the AcMNPV baculovirus p10 promoter, flanked by sequences for homologous recombination at the polyhedrin locus. The plasmid pΔPhC3T-N was used with a baculovirus DNA linearized at the polyhedrin locus (Agate bioservices, Bagard, France) for cotransfection in Sf9 cells to obtain the recombinant viral clone, Bac-N to produce the N nucleoprotein.

For the coproduction of N, G_N_ and G_C_ proteins (N- G_N_/G_C_) coding-sequences were coexpressed in a single baculovirus vector. When produced alone, the nucleoprotein N forms complex multimeric structures that were isolated from infected cell supernatants. However, when N and G_N_ /G_C_ were coexpressed, virus like particles (VLPs) were generated and shown to contain the correctly processed G_N_ /G_C_ precursor [29], and to form spherical structures with projections protruding from the surface, which mimics RVF viral particles, with the G_N_ and G_C_ proteins required for the stable morphology of the VLP structures. Hence, for the production of monoclonal antibodies, we decided, to coexpress N and G_N_/G_C_ sequences, assuming that together, these antigens would fold correctly. For this purpose, pTenTwin plasmid vector (Agate bioservices, Bagard, France) with tail-to-tail dual p10 promoters was used. The N sequence was amplified using the primer Nrif5’HindIII (5’- nnnnAAGCTTACCATGGACAACTATCAAGAGC-3’) and the primer Nrif3’BglII (5’-nnnnAGATCTTTAGGCTGCTGTCTTGTAAGC-3’), and inserted at the HindIII-KpnI sites under control of a first p10 promoter. The G_N_/G_C_ sequence was amplified using G_N_Gc5’BamHI (5’-nnnnGGATCCACCATGGCAGGGATTGCAATG-3’) and G_N_Gc3’NotI (5’-nnnnGCGGCCGCTTATGAGGCCTTCTTAGTGG-3’) primers and inserted at the BamHI-NotI sites under the control on a second p10 promoter. The dual vector obtained, pTenTwin-N/ G_N_Gc, was used for cotransfection with the BacTen baculovirus DNA [30] to generate a recombinant viral clone, BacTen-N/ G_N_Gc, coexpressing the N and G_N_/G_C_ sequences at the p10 locus.

#### Production and purification of the recombinant N and N-G_N_/G_C_

Insect cells have been shown to be suitable for the production of RVFV proteins [30]. Bac-N and BacTen-N/G_N_/G_C_ recombinant baculoviruses were amplified to generate high-titer stocks in Sf9 cells grown in HyQ-SFX serum-free medium (GE Healthcare, France). For production, a total of 1.10^9^ Sf9 cells was infected at a MOI of 3, supernatants were collected at day 3 post-infection. The RVF N nucleoprotein and the N-G_N_/G_C_ coexpressed proteins were purified mainly as described by Liu [29]. Briefly supernatants containing either recombinant baculovirus N or N-G_N_/G_C_ were clarified by centrifugation for 30 minutes and precipitated by ultracentrifugation through a cushion of 20% sucrose. N was further purified by size exclusion chromatography, and N-G_N_/G_C_ coexpressed proteins were submitted to a potassium tartrate-glycerol gradient. Cells, supernatants and purified N-G_N_/G_C_ were analyzed by SDS-PAGE followed by Western blot. Briefly, Western blot membranes were incubated either with RVF mouse anti-N (ID.vet, France) or anti-N/G_N_/G_C_ (RVF polyclonal bovine hyper immune serum, Mayotte 2008) primary antibodies, followed by either rabbit or bovine alkaline phosphatase-conjugated secondary antibodies and chemical detection.

#### Production and selection of monoclonal antibodies (Mabs)

N-G_N_/G_C_ antigens coproduced and copurified as described above were administered intraperitoneally to five OF1 mice at the dose of 100 μg/mouse at days 0, 21 and 41. At day 63, a final boost was carried out three days before spleen cells were fused with SP2/0 myeloma cells at a ratio of 1:5 in the presence of polyethylene glycol 1500 (PEG, Sigma Aldrich, France). Hybridoma cells were selected in DMEM medium (Sigma Aldrich, France) containing the hypoxanthine-aminopterin-thymidine (HAT) selective medium (Sigma Aldrich, France), fetal bovine serum, 15% (Life Technologies Gibco, France), hybridoma enhancing supplement macrophage-like origin (HES), 1% (Sigma Aldrich, France), L-glutamine, 200mM (Life Technologies, Gibco, France), penicillin, 10,000 units/streptomycin, 10 mg/ml (Life Technologies, Gibco, France). Stepwise the HAT medium was replaced by hypoxanthine-thymidine (HT) medium (Sigma Aldrich, France) followed by maintenance medium (Biotem, Le Rivier d’Apprieu, France). The hybridoma clone supernatants were screened and selected using three separate successive tests for their reactivity against RVFV (i) a first screening test, an indirect ELISA with RVFV coated N-G_N_/G_C_ antigens at a concentration of 1 μg/ml, and peroxidase-conjugated goat anti-mouse IgG/IgM detection antibody diluted 1:10000 (Jackson ImmunoResearch, USA), the positive cut-off value was an OD (Optical Density) > 0.300, and the OD value of the positive control > 1.2 (ii) an immunofluorescence assay (IFA) based on Vero cells infected with the RVFV Smithburn strain at a multiplicity of infection (MOI) of 0.25 pfu (particle forming unit) per cell with an anti-mouse FITC, diluted 1:1000 (Dako, France), as detection antibody. The presence of green fluorescence was considered as positive, Mab directed against peste des petits ruminants virus (PPRV) (gift from G. Libeau, CIRAD, France) as negative control and (iii) an ELISA based on the Bac-N nucleoprotein expressed by Sf9 insect cells with peroxidase-conjugated goat anti-mouse diluted 1:10000 (Jackson ImmunoResearch, USA), as detection antibody, the positive cut-off value being an OD value > 0.300, and the OD value of the positive control, Mab anti-N (ID.vet, Grabels, France) > 1.2.

#### Mabs selection and characterization

After fusion of splenocytes isolated from a mouse immunized with copurified N-G_N_/G_C_ proteins of RVFV with cells of the murine myeloma line SP2/0, seven hybridoma lines exhibiting high reactivity against the protein N of RVFV and belonging to the IgG subclass IgG1 or IgG2a following three rounds of cloning were selected and their suitability for RVFV antigen detection in the LFT was investigated.

#### Conjugation of Mab 8E10-4A4 to gold micro particles

The Mab 8E10-4A4 of IgG1 isotype was selected and coupled with 40 nm colloidal gold particles. Briefly, the Mab was dialyzed in a 20mM Borat-Borax buffer at pH 7.3 and then mixed with an EDTA-NHS chelator prior to conjugation. Colloid gold particles (40nM) were mixed in UPH-water and activated by adjusting the pH to 7.1 with 100mM Na_2_CO_3_ buffer. The Mab/chelate solution was slowly added to the colloid gold solution under thorough mixing. The conjugation reaction was stopped by adding a blocking/stabilizer solution also under thorough mixing. The conjugate solution was then centrifuged at +8°C, 14000 g for 20 minutes. The supernatant was removed and the pellet dissolved with a gold conjugation buffer, subsequently filtered through 0.22μm filter and stored at +4 °C until applied to the nitrocellulose membrane.

#### Adsorption of Mab 10H3-4E4-3D5 to nitrocellulose

The Mab 10H3-4E4-3D5 of IgG2a isotype, dissolved in phosphate buffer 10 mM, NaCl 0.15 M, pH7.4 at a final concentration of 2.2 mg/ml was applied to the nitrocellulose membrane (Hi-Flow^™^ Plus HFC 13504 membrane, Millipore, USA) using Bio-Dot air-brush equipment (Bio-Dot, UK). Fifty microliters of the Mab solution were added per 30 cm of membrane. Rabbit anti-mouse antibodies (Dako, Denmark) were applied (control band) at a concentration of 2.2 mg/ml parallel to the Mab line (test band). The membranes were dried at 37°C for 45 min and stored in sealed foil sachets until use.

#### Adsorption of gold/Mab 8E10-4A4 conjugate antibody to filters

The Mab 8E10-4A4 gold conjugate of IgG1 isotype was applied to the freagent pads (Whatman 17 CHR, UK) using Bio-Dot air-brush equipment (Bio-Dot, UK), at a volume of 1 μl/mm filter. The reagent pads were dried.

### Design and lateral flow device test (LFT) procedure

The test strip was constructed on the principles of immunochromatography using colloidal-gold-labeled Mabs. We used the two Mabs generated against the N protein of RVFV described above: the Mab 8E10-4A4 gold conjugate and the Mab 10H3-4E4-3D5. Mab 10H3-4E4-3D5 was immobilized onto a nitrocellulose membrane for the test line zone and rabbit anti-mouse antibodies (Dako, Denmark) were immobilized for the control line zone to capture unbound Mab. A reagent pad containing colloidal gold-labeled Mab 8E10-4A4 was located in front of the sample hole and overlaid onto the base of the nitrocellulose membrane, parallel to the control and the antibody bands, stuck to the membrane with adhesive cut into 0.8 cm wide strips, assembled in a device as described previously [31]. Aliquots (150 μl) of either viral isolates mimicking clinical specimens, or viral supernatants diluted in DMEM, or serum samples were mixed with an equal volume of LFT sample buffer (0,1% Casein, 20 mM Borat-Borax buffer, 0,5% Tween 20) and the mixture was applied to the sample pad (S). This resulted in rehydration of the air-dried conjugated gold Mab and and their migration by capillary action along the membrane. If RVFV antigen was present in the sample then the RVFV-Mab-conjugate complex was captured by the immobilized Mab on the membrane at the ‘T’ (test) line and resulted in their accumulation, which could be visualized as a red line to signify a positive result. Excess (or unbound) Mab-labelled gold particles continued to migrate along the device until being captured by the immobilized rabbit anti-mouse antibody and the formation of a red ‘C’ (control) line, to validate the test. The test (T) and control (C) lines were checked for the development of color after 10 minutes and again after 30 minutes as it might take longer time for weak positives to form a visual band scored subjectively from negative to strong.

### Assessment of the diagnostic and analytical sensitivity ((DSe/ASe) and of the diagnostic and analytical specificity (DSp/Asp)

According to the OIE guidelines [32], estimates of DSe (proportion of samples from known infected reference animals that test positive in an assay) and DSp (the proportion of samples from known uninfected reference animals that test negative in an assay) are the primary performance indicators established during validation of an assay. Analytical specificity (ASp) defined as the ability of the assay to distinguish the target analyte (e.g. a viral antigen) from non-target analytes, including matrix components and analytical sensitivity (ASe) defined as the estimated amount of analyte in a specified matrix that would produce a positive result at least a specified percent of the time are the first steps of the validation of an assay. The limit of detection (LOD) is a measure of the ASe. Although RVFV is considered as a single genotype and serotype, diagnostic sensitivity (DSe) was assessed with sera spiked with different RVF viral isolates from different geographical origins and collected over a period of 69 years to mimic clinical specimens (serum or fluids from aborted fetuses) (n = 25, Table 1A) and sera of the ongoing RVF outbreak occurring on Mayotte [10] (n = 10, Table 1A).

**Table 1:** Origin and results of the samples used to assess diagnostic sensitivity and specifity of the RVF LFT. Identification, year of isolation, origin of virus strains or samples of sera tested in this study.

A. Sensitivity					
Genus	Strain/Species	Year of isolation	Country of origin	Source	LFT result
					(N° pos/total N°)
Phlebovirus	RVFV strain Smithburn	1944	Uganda	mosquito	Positive (1/1)
Phlebovirus	RVFV strain Lunyo	1955	Uganda	mosquito	Positive (1/1)
Phlebovirus	RVFV strain AN 1830	1956	South Africa	sheep	Positive (1/1)
Phlebovirus	RVFV strain KEN56/B2653/IB8	1963	Kenya	bovine	Positive (1/1)
Phlebovirus	RVFV strain 56/74	1974	South Africa	cow	Positive (1/1)
Phlebovirus	RVFV strain 252/75	1975	South Africa	NA	Positive (1/1)
Phlebovirus	RVFV strain ZH501	1977	Egypt	human	Positive (1/1)
Phlebovirus	RVFV strain VRL688/78	1978	Zimbabwe	bovine	Positive (1/1)
Phlebovirus	RVFV strain AR 20368	1981	South Africa	mosquito	Positive (1/1)
Phlebovirus	RVFV ARD 38388	1983	Burkina Faso	mosquito	Positive (1/1)
Phlebovirus	RVFV ARD 38661	1983	Senegal	mosquito	Positive (1/1)
Phlebovirus	RVFV 143/83	1983	Namibia	human	Positive (1/1)
Phlebovirus	RVFV ANK 6087	1984	Guinea	bat	Positive (1/1)
Phlebovirus	RVFV SPU45/85	1985	Zambia	human	Positive (1/1)
Phlebovirus	RVFV SPU204/855	1985	Angola	human	Positive (1/1)
Phlebovirus	RVFV An991	1991	Madagascar	bovine	Positive (1/1)
Phlebovirus	RVFV Tambul	1994	Egypt	ovine	Positive (1/1)
Phlebovirus	RVFV SPU12/98/2	1998	Somalia	goat	Positive (1/1)
Phlebovirus	RVFV strain AR21229	2000	Saudi Arabia	mosquito	Positive (1/1)
Phlebovirus	RVFV F057	2007	Kenya	human	Positive (1/1)
Phlebovirus	RVFV AL51	2009	Madagascar	mosquito	Positive (1/1)
Phlebovirus	RVFV HA 09–001	2008	Madagascar	bovine	Positive (1/1)
Phlebovirus	RVFV strain 26010 YK0	2010	Mauritania	camel	Positive (1/1)
Phlebovirus	RVFV strain MRU 2687	2013	Mauritania	goat	Positive (1/1)
Phlebovirus	RVFV strain SN 2	2013	Senegal	goat	Positive (1/1)
Phlebovirus	Z_LD_8023	2019	Mayotte, France	ovine	Positive (1/1)
Phlebovirus	Z_LD_8024	2019	Mayotte, France	ovine	Positive (1/1)
Phlebovirus	Z_LD_2700/5	2019	Mayotte, France	bovine	Positive (1/1)
Phlebovirus	Z_LD_8016	2019	Mayotte, France	bovine	Positive (1/1)
Phlebovirus	Z_LD_8107	2019	Mayotte, France	bovine	Positive (1/1)
Phlebovirus	Z_LD_8248	2019	Mayotte, France	bovine	Positive (1/1)
Phlebovirus	Z_LD_8627	2019	Mayotte, France	bovine	Positive (1/1)
Phlebovirus	Z_LD_8656	2019	Mayotte, France	bovine	Positive (1/1)
Phlebovirus	Z_LD_8657	2019	Mayotte, France	bovine	Positive (1/1)
Phlebovirus	Z_LD_Liver 160119	2019	Mayotte, France	bovine	Positive (1/1)
**B. Specificity**					
Phlebovirus	Arumowot virus	1963	Sudan	Culex antennatus	Negative (0/1)
Phlebovirus	Gabek forest virus	1981	Unknown	Unknown	Weak band (1/1)
Phlebovirus	Saint Floris virus	1981	Unknown	Unknown	Negative (0/1)
Flavivirus	Dengue virus, serotype 2	2012	Reunion Island	human	Negative (0/1)
Orthobunyavirus	Akabane virus	1978	Unknown	Unknown	Negative (0/1)
Orthobunyavirus	Shamonda virus	1972	Unknown	Unknown	Negative (0/1)
Alphavirus	Chikungunya virus Chik #4	2006	Reunion Island	human	Negative (0/1)
Reovirus	Bluetongue virus, serotype 2	2002	Corsica, France	ovine	Negative (0/1)
Morbillivirus	PPRV 75/1	1975	Nigeria	goat	Negative (0/1)
NA	Sera samples	2017	Mainland France	goat/bovine	Negative (1/31)
NA	Sera samples	2015	Union of Comoros	goat/bovine	Negative (0/20)
NA	Sera samples	2016	Reunion Island	bovine	Negative (0/30)
NA	Sera samples	2010	Tunisia	goat/camel	Negative (0/30)
NA	Sera samples	2011	Madagascar	goat/bovine	Negative (0/20)
NA	Sera samples	2016	Mayotte, France	bovine	Negative (0/19)
NA	Sera samples	2019	Mayotte, France	bovine	Negative (0/10)

NA stands for Not Applicable, N° for Number and PPRV for Peste des Petits Ruminants Virus

Arboviruses belonging to the *Phlebovirus* genus and viruses belonging to other viral genera but producing the same clinical features in humans and/or ruminants (i.e. flaviviruses, alphaviruses) (n = 9, Table 1B) were tested to assess diagnostic specificity (DSp), i.e. the proportion of samples from known uninfected reference animals that test negative in the assay, as they could be considered as the source of possible cross-reactions in diagnostic assays. In addition, animal serum samples known to be seronegative for RVF of different origins (mainland France, Tunisia, Reunion Island, Mayotte, Union of Comoros, and Madagascar) (n = 169, Table 1B) were used for this evaluation.

To determine ASe, eight titrated suspensions from RVFV of different origins (Uganda, Madagascar, Mauritania and South Africa) adapted to cell culture were selected and diluted in RVF negative sheep sera to determine the limit of detection (LOD) of the LFT using 10-fold serial dilutions in RVFV negative cattle serum (Life Technologies, Gibco, France) mimicking clinical specimens. A volume of 150 μL of each of the dilutions was tested. Samples were scored as true positives when detected positive with the real-time RT-PCR technique [16], which was considered as the gold standard in our study.

## Results

### Design of the lateral flow test (LFT)

#### Production and purification of the recombinant N and N-G_N_/G_C_

**Fig 1:**
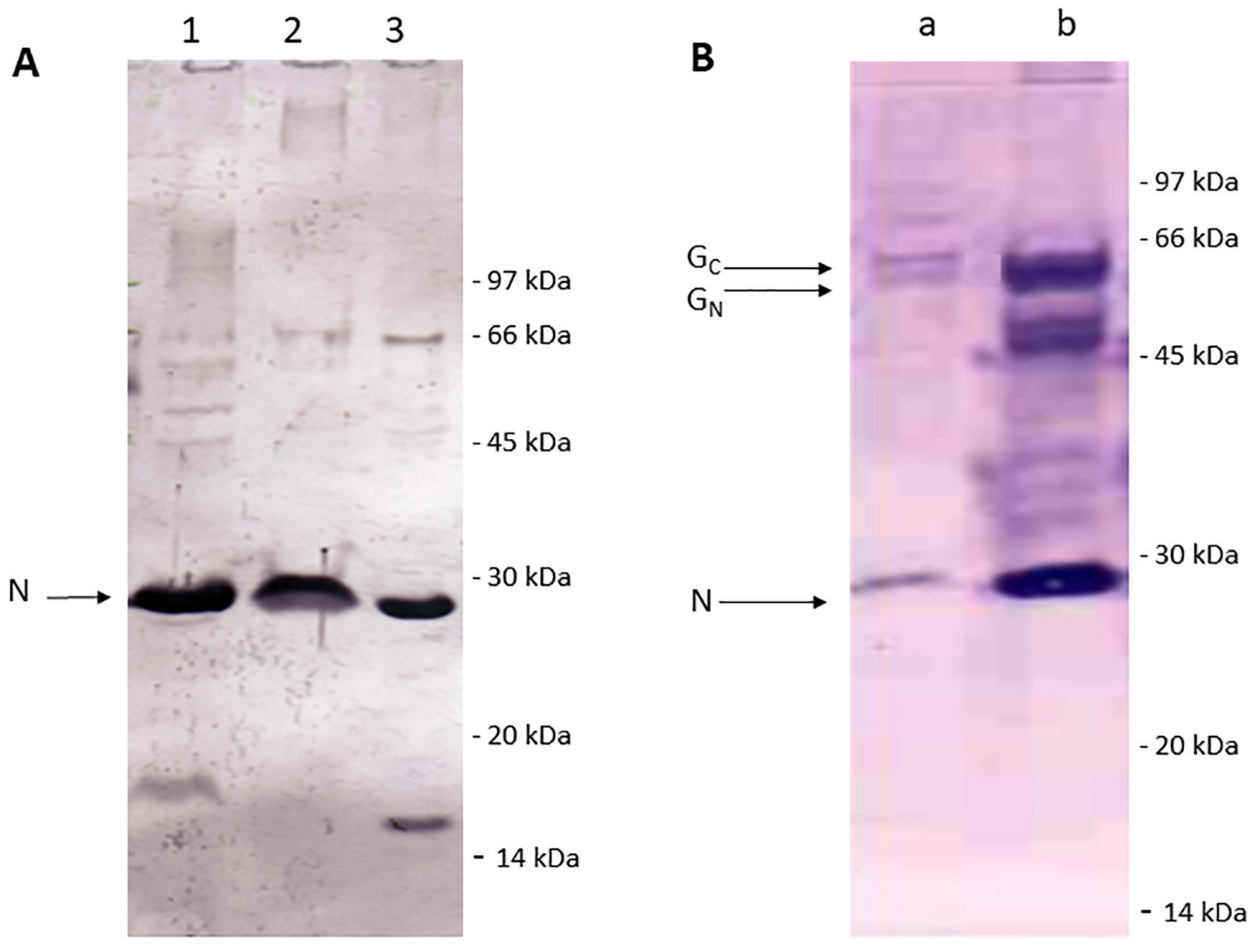
Coproduction of RVFV N and G_N_/G_C_ proteins in insect cells. Controls (uninfected and wild-type AcMNPV-infected cells) gave no signal. A. SDS-PAGE/Western blot, anti-N detection. Lane 1: Sf9 crude cell lysates coproducing N and G_N_/G_C_ proteins. Lane 2: Sf9 crude supernatant coproducing N and G_N_/G_C_ proteins. Lane 3: purified N and G_N_/G_C_ proteins. B. SDS-PAGE/Western blot, anti-N/G_N_/G_C_ detection. Lane a: Sf9 crude supernatant coproducing N and G_N_/G_C_ proteins. Lane b: copurified N/G_N_/G_C_ proteins. A double band at/above 45 kDa might correspond to products of G_N_/G_C_ cleavage and maturation, and possibly to partial and/or nonspecific processed forms.

RVFV N or N-G_N_/G_C_ proteins coproduced in insect cells were analyzed by SDS-PAGE followed by Western Blot incubated either with RVF mouse anti-N (Fig 1A) or anti-N/G_N_/G_C_ antibodies (Fig 1B). Western blot analysis using monoclonal antibody specific to RVFV N protein revealed a strong band of 26 KDa equivalent to the expected size of the N protein in the infected cell lysate, supernatant and in its purified form (Fig 1A, lane 1, 2 and 3 respectively). Western blot analysis using RVF polyclonal bovine hyper immune serum known to recognize N/G_N_/G_C_ proteins detected 3 bands at respectively 26 kDa (N protein),55 kDa (G_N_ protein) and 58kDa (G_C_ protein) in the crude supernatant and as copurified proteins (Fig 1B, lane a and b). A double band at/above 45 kDa might correspond to products of G_N_/G_C_ cleavage and maturation, and possibly to partial and/or nonspecific processed forms.

#### Mab selection and characterization for use in the LFT

**Fig 2:**
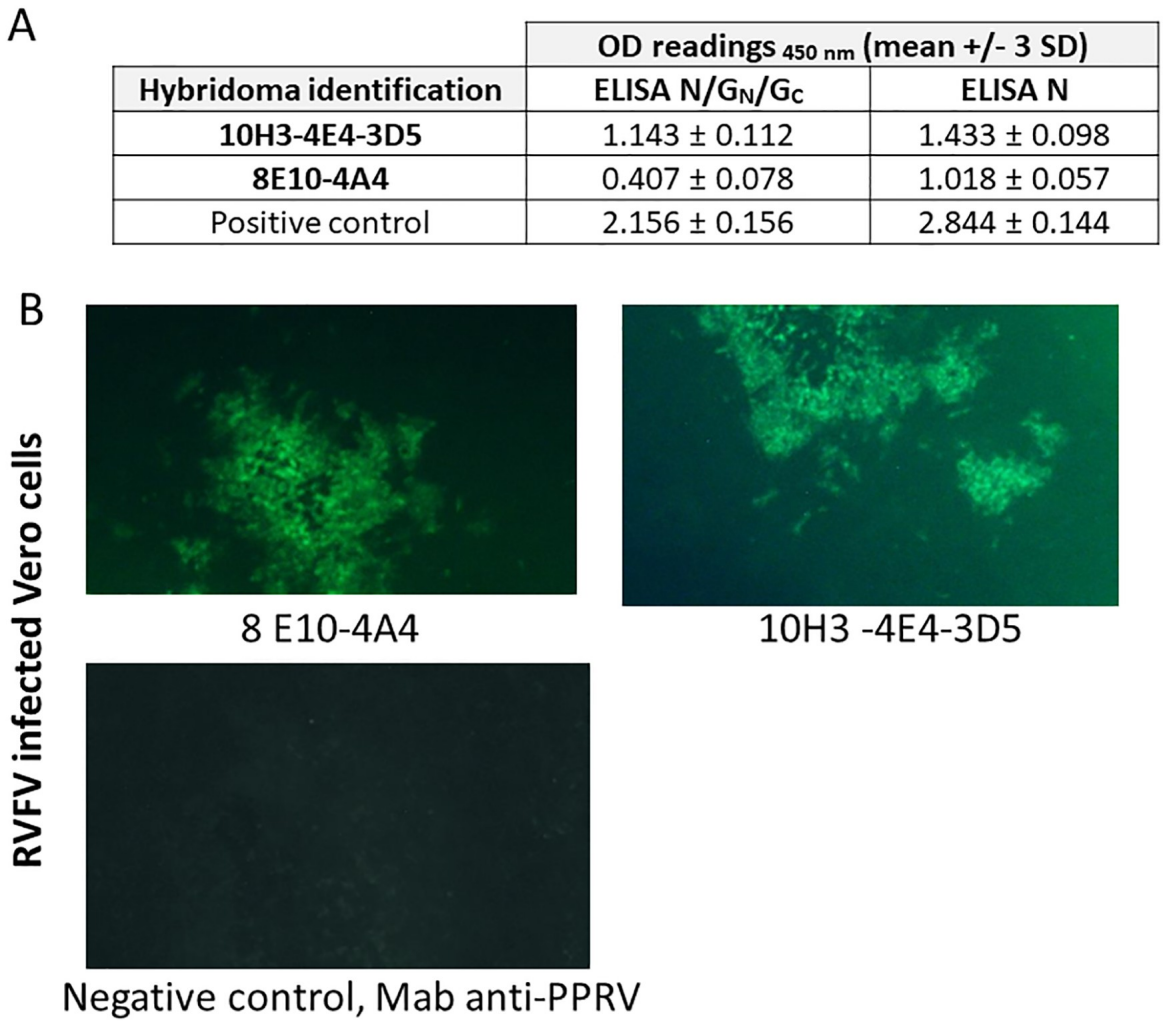
Specificity of the two Mabs 8E10-4A4 and 10H3-4E4-3D5 used in combination in the LFT for RVFV. Specific binding of the Mabs to RVFV N protein was examined by (A) ELISA with RVFV copurified N/G_N_/G_C_ proteins (ELISA N/G_N_/G_C_), ELISA with coating of Bac-N nucleoprotein expressed by Sf9 insect cells (ELISA N). Readings are OD (Optical Density) values measured at 450 nm. Mabs were tested diluted 1:10 in culture medium and run in duplicates (mean +/- 3 SD). The positive cut-off value of the test is an OD value > 0.300, the positive control (mouse immunized with RVF N/G_N_/G_C_ sampled at day 41, dilution 1:100) must have an OD value > 1.200 and the negative control (non immunized OF1 mouse control) must have an OD value < 0.300. A tested sample is detected positive when the OD value > 0.300. (B) Immunofluorescence Assay (IFA) x 10, green fluorescence: positive sample, Mab directed against peste des petits ruminants virus (PPRV), negative control, no fluorescence.

Based on the results of the three separate tests previously described, two purified Mabs, Mab 10H3-4E4-3D5 as coating Mab and Mab 8E10-4A4 as the conjugate Mab were tested in combination at different concentrations based on experimental results and chosen for further sensitivity and specificity validation (Fig 2).

#### Design of in the LFT

**Fig 3:**
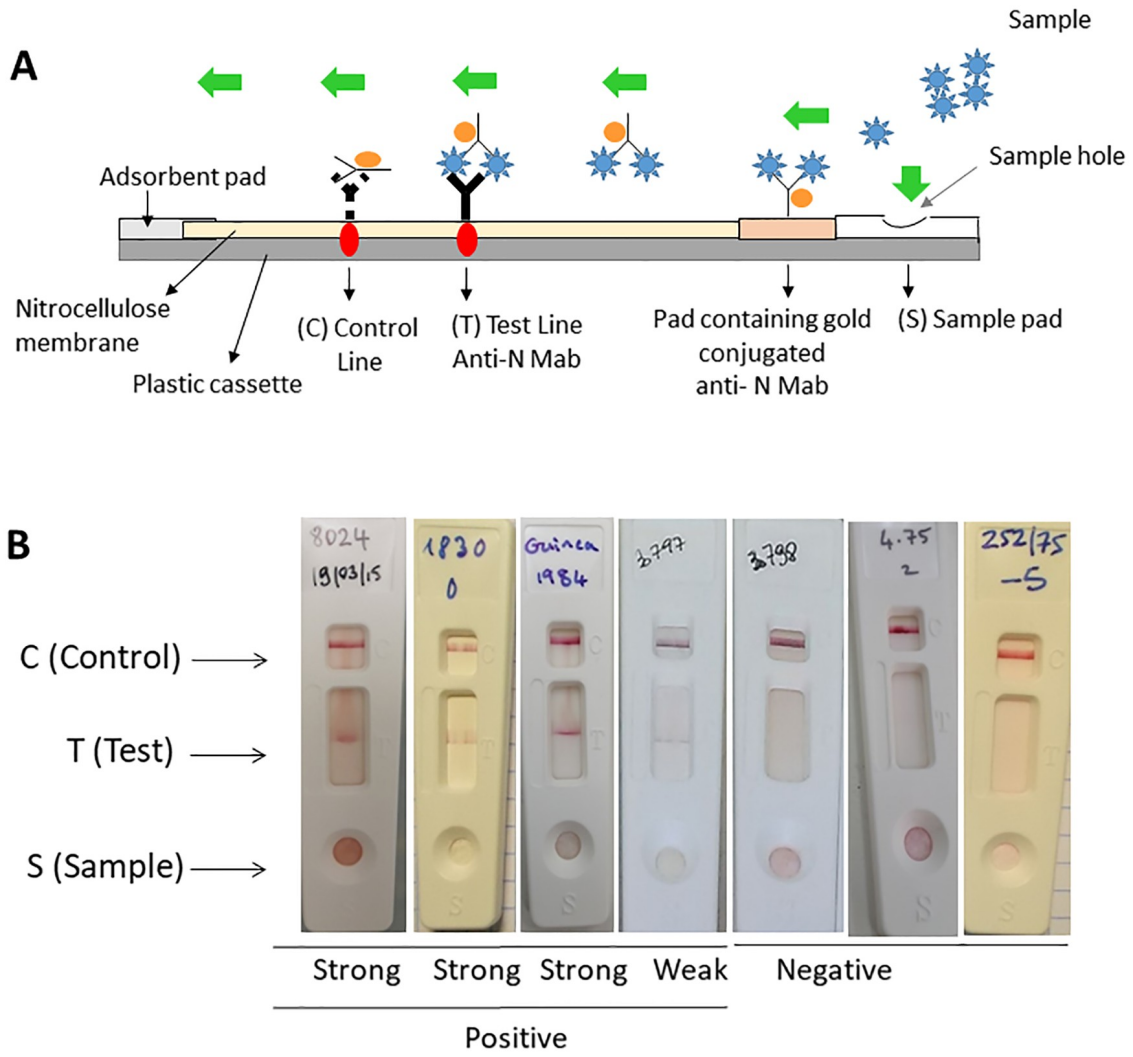
RVF LFT strip test for the detection of RVF infection using the two selected Mabs. (A) Diagram of the rapid RVF LFT for the detection of the RVF N protein, (B) Results of LFT strip. The serum sample or the viral suspension (150 μl) was mixed with 150 μl of sample buffer and applied to the S hole of the strips for migration. Results were recorded after 15 minutes. The test is valid when a red band is visible at the same level as the label C. S stands for Sample, C for Control and T for Test.

In a positive sample, the RVFV-Mab-conjugate complex captured by the immobilized Mab on the membrane corresponds to a solid red line at the ‘T’ (test) of the device. A red ‘C’ (control) line always appear and validate the test (Fig 3A). LFT strips showing negative, weak and positive results are shown in Fig 3B.

### Estimates of diagnostic sensitivity (DSe) and diagnostic specificity (DSp)

#### Diagnostic sensitivity of the LFT (DSe)

A total of 25 isolated strains mimicking clinical specimens of different geographical origins and 10 clinical samples originating from the ongoing outbreak of Mayotte (2019) [10] detected RVF positive by a Taqman RT-PCR technique which is considered as the current reference detection system [16] were also detected positive by the LFT (Table 1A) giving a DSe of 100% (CI 95%[90,1; 100]) (n = 35).

#### Diagnostic specificity (DSp) of the LFT

Diagnostic specificity (DSp) was assessed on 160 serum samples known to be negative for RVFV by seroneutralization test and cELISA but collected in tropical countries (Madagascar, Comoros, Mayotte) where RVF has been circulating to detect possible cross reactions or in other areas where RVF introduction is considered at risk (Tunisia, mainland France, Reunion Island). Only one sample among 160 originating from mainland France was found positive with RVF LFT but negative with the gold standard reference test (n = 160, Table 1B). In addition, nine vector-borne viruses or viruses that produce clinical signs similar to RVF, such as abortion or fever, were tested (n = 9, Table 1B) and were detected negative for RVF by LFT except for the Gabek forest virus that produced a weak band. Finally, the test gave a DSp of 98.81% (CI 95% [95.8; 99,7]) (n = 169) (Table 2).

**Table 2:** Analytical specificity (ASp). Agreement between the RVF LFT results and the PCR results [16].

		**PCR results**		
		Positive	Negative	*Total*
**LFT results**	Positive	35	2	37
	Negative	0	167	167
	*Total*	35	169	204

#### Determination of the limit of detection (LOD) of the LFT, a measure of ASe

Eight titrated viral suspensions from RVFV originating from different countries (Uganda, Madagascar, Mauritania and South Africa) (Table 3) were used spiked in RVF negative sera to detect the LOD, a measure of ASe. The lowest numbers of pfu detected with this assay were 2.3 x10^3^ pfu, 3x10^3^ pfu and 9x10^3^ pfu for the South African AN1830, the Smithburn and the Mauritanian 26010-YK0 strains respectively.

**Table 3:** Analytical sensitivity (ASe) (estimated limit of detection (LOD)) based on 8 titrated RVF strains.

Virus strain	Original host	Location	Initial virus concentrations (pfu/ml)*	Limit of LFT detection (pfu)**
RVFV strain 56/74	cow	South Africa	1x10^7^	1.5x10 ^5^
RVFV strain 252/75	nd	South Africa	7x10^6^	1.04x10^4^
RVFV strain Ar 20368	mosquito	South Africa	1.5x10^7^	2.2x10 ^5^
RVFV strain AN 1830	sheep	South Africa	1.4x10^7^	2.2x10^3^
RVFV strain AL51	mosquito	Madagascar	4.1x10^7^	6.1x10^4^
RVFV strain HA 09–001	bovine	Madagascar	1.8x10^7^	2.7x10^5^
RVFV Strain 26010-YK0	camel	Mauritania	6.3x10^6^	9.4x10^3^
RVFV strain Smithburn	mosquito	Uganda	2x10^6^	3x10^3^

* pfu/ml, particle forming units per ml,

**pfu deposited on the strip

nd, not determined

## Discussion

Recent outbreaks in several countries including Sudan, Uganda, Niger, Mali (2017–2018), Kenya and Mayotte (2018–2019) confirm the active circulation of the disease. Human and animal movements in territories facing rural movements and civil wars are likely to facilitate RVFV spread and its extension outside its traditional boundaries towards northern Africa [33] and possibly to Europe. In European countries, currently free of RVFV, its introduction could cause severe outbreaks in naïve human and/or animal populations. As RVF is a vector borne disease, the threat is also increasing due to global warming leading to (i) an increase in mosquito density and diversity, (ii) vector competence being acquired by new species [2, 5, 34]. The development of rapid diagnostic tools able to detect RVF infection is crucial for both human and animal health monitoring in endemic and at-risk areas for RVF. The aim of our study was (i) to develop and evaluate a prototype of a rapid and robust RVF LFT and (ii) to investigate its intrinsic parameters. As it is the case with viruses that are members of the *Bunyavirales* order, the N nucleocapsid protein was shown to be a key immunogenic protein that induces a strong immune response [35–36]. Antibodies against the nucleoprotein N are readily detected early after infection and in convalescent individuals, providing a robust basis for detection and diagnostic of the disease. ELISA diagnostic tests have been developed based on this antigen and produced promising results [11, 37]. This suggests the nucleoprotein N is a highly suitable target antigen for the detection of RVFV infection. In the present study, RVFV specific Mabs were produced against the N nucleoprotein and conjugated with colloidal gold to bind RVFV antigens captured by immobilized membrane to form a red band, which indicates the presence of RVFV antigens. The performance of the LFT we developed for detection of RVF infection was evaluated on sera spiked with known positive viral suspensions mimicking clinical specimens (n = 25) as well as viremic clinical samples of an ongoing outbreak (Mayotte 2019) (n = 10) and known RVF negative sera of different origins (Tunisia, Madagascar, Mayotte, Reunion Island, Union of Comoros, mainland France). The RVF LFT was able to detect all 35 RVFV spiked or clinical samples from different geographical regions. Specificity was determined to be 98.81%, (CI 95% [95.8; 99,7]) based on (i) testing known negative animal serum samples from different countries (n = 160) and (ii) vector-borne viruses or viruses giving clinical signs similar to RVF (n = 9). A weak positive band was detected when testing the Gabek forest virus (GFV). This could be explained by the high percentage of homology observed between the segments of RVFV and GFV illustrated by the closeness of the clade RVFV and GFV [38]. These preliminary data need to be strengthened with additional field serum samples originating from ongoing outbreaks around the world, even though the quantification of the viral load present in serum field samples is hard to perform due to the very few laboratories able to handle advanced cell culture and molecular biology experiments located where the outbreaks occur. Comparison with other diagnostic techniques will help in that quantification. The limit of detection of this LFT was investigated using eight titrated suspensions of RVFV spiked in dilution in RVF negative sheep sera. The values varied among the isolates and ranked from 10^3^ to 10^5^ pfu. Previous ELISA based on RVF antigens were found to be less sensitive since they are able to detect 10^2.2^ to 10^3.2^ TCID_50_/reaction volume [37]. Viremia becomes demonstrable in one-week-old lambs within 16 hours of peripheral infection and persists for the duration of the illness for at least 36 to 42 hours [39]. In older sheep, goats and cattle, viremia becomes demonstrable one to two days after infection and persists for seven days with a peak from day 2 to day 5. Observed viremia ranged from 10^5.6^ to 10^9^ of mouse median lethal doses (MIPLD_50_) per ml in naturally infected domestic animals up to 10^8.6^ in humans [40]. Maximum viremia recorded were 10^10.1^ MIPLD_50_/ml in lambs, 10^7.6^ in sheep [41], 10^7.5^ in calves, and 10^5.6^ in goats [42]. Viremia titers in experimentally infected goats range from 10^4^ to 10^6.5^ log_10_ RVF copies/ml [43–44]. Viremia of lower intensity and shorter duration has been detected in other animal species that have been studied, but quantitative data are difficult to obtain. In adult African buffalo and ponies, viremia was recorded at 10^5.4^TCID_50_ /ml and 10^2.5^MIPLD_50_/ml respectively [45–46].

The original aim of the LFT was to detect RVF infection in the field in serum samples in cases of suspected outbreaks. In that context, the viremia expected in infected animals is likely to be high with titers above 10^5^ pfu irrespective of the species of domestic animal tested (goats, sheep, cattle, camels). In that sense, the level of specificity obtained for the LFT would be high enough to test a field sample for a rapid first detection of initial outbreaks (which would still require confirmation of negative or positive results by a reference laboratory) or in the case of disease surveillance in the framework of a control programme when RVF has already been confirmed. The level of detection of the LFT could be improved either by experimenting with the addition of a secondary antibody on top of the conjugated Mab in the construction of the cassette or by testing several types of membranes. Alternative colloid gold conjugation methods or other labels could also be evaluated to improve the sensitivity. Recovery of viral RNA from the same type of LFT membrane has been already proven for other viruses such as foot and mouth disease virus [47–48] and is another great outcome of this type of diagnosis, specifically for viruses spreading in areas where access to diagnostic facilities is limited. The type of samples that have been validated in this study is serum samples as it could be easily used in a field setting for early RVF detection. Comparison of whole blood versus serum, testing of long-term storage conditions are worth to be evaluated in future trials in order for this test to be field deployable to locations where this will ultimately be needed. The use of RVF LFT by trained personnel wearing the highest level of protective clothing and using appropriate equipment will provide rapid and objective support to veterinarians in their clinical judgment of the disease and also help dispatch field materials to national or international reference laboratories for confirmation. The sooner the disease is diagnosed, the sooner the appropriate measures can be taken. The use of this first-line LFT should accelerate the start of epidemiological investigations in the case of RVF outbreaks. Furthermore, the difficulties and the high cost of sending infectious material from rural veterinary or district health facilities, where the disease often occurs, to the regional or national reference laboratory and the biosecurity risk it presents underline the need for a thermo-stable, non-infectious standard mode of transporting diagnostic samples. The cost of the LFT itself and its shipment to regional facilities versus saving on expenses for standard sample collection and transportation is likely to vary from country to country and depends on the supply and demand as well as the competitive offers of the private companies able to produce it. Therefore the cost implications and the potential for this LFT to be easily affordable in low and limited resource settings needs to be taken into consideration in the long-term implementation of this LFT.

### Conclusion

The performances of the RVF LFT are promising for field use, where the test could help to establish rapid preliminary diagnostic results particularly in suspected cases in the field, which would then have to be confirmed using WHO (World Health Organization) or OIE (World Organisation for Animal Health) recommended tests at central laboratories. The specificity and sensitivity of the evaluated test are lower than the ones of molecular-based techniques (LAMP, PCR) but are adequate for specific rapid initial detection of RVF outbreaks or disease surveillance in control programmes. However, there is still room for improvement in LFT performances by changing several parameters i.e. the sample buffer, the type of membrane or the addition of secondary labeled antibodies. This rapid and easy RVF LFT device does not require special laboratory equipment but does require trained staff wearing appropriate biosecure protective clothing. Although the Lateral Flow Test (LFT) is easy to use for non-laboratorians outside a biosafety containment reference laboratory normally used for RVF testing, it nevertheless requires appropriate training to avoid any accidental infection during the removal and handling of a potentially infected sample of body fluid (serum being the ideal type of sample in the case of RVF outbreak or fluid from aborted fetuses) for LFT testing. LFT can be performed in the field where epizootics occur. RVF LFT will be particularly valuable in remote areas or in territories where there are no diagnostic facilities but it is important to underline that staff needs to be trained to handle it safely under the highest possible biosecurity conditions.
